# Climatic Factors and Community — Associated Methicillin-Resistant *Staphylococcus aureus* Skin and Soft-Tissue Infections — A Time-Series Analysis Study

**DOI:** 10.3390/ijerph110908996

**Published:** 2014-08-29

**Authors:** Krushna Chandra Sahoo, Soumyakanta Sahoo, Gaetano Marrone, Ashish Pathak, Cecilia Stålsby Lundborg, Ashok J. Tamhankar

**Affiliations:** 1 Department of Public Health Sciences (Global health/IHCAR), Karolinska Institutet, Stockholm 17177, Sweden; E-Mails: sahookrushna@yahoo.com (K.C.S.); gaetano.marrone@ki.se (G.M.); Cecilia.Stalsby.Lundborg@ki.se (C.S.L.); ejetee@gmail.com (A.J.T.); 2 Department of Microbiology, Kalinga Institute of Medical Sciences (KIMS), Super Religare Laboratories Limited, Kalinga Hospital, Bhubaneswar 751024, India; E-Mail: sk.soumya@gmail.com; 3 Department of Paediatrics, R.D. Gardi Medical College, Ujjain 456006, India; 4 Department of Women and Children’s Health, International Maternal and Child Health Unit, Uppsala University, SE 751 85 Uppsala, Sweden; 5 Indian Initiative for Management of Antibiotic Resistance (IIMAR), Department of Environmental Medicine, R.D. Gardi Medical College, Ujjain 456006, India

**Keywords:** skin and soft tissue infections, *Staphylococcus aureus*, methicillin-resistant *Staphylococcus aureus* (MRSA), temperature, relative humidity, time-series analysis, antibiotic susceptibility testing

## Abstract

Skin and soft tissue infections caused by *Staphylococcus aureus* (SA-SSTIs) including methicillin-resistant *Staphylococcus aureus* (MRSA) have experienced a significant surge all over the world. Changing climatic factors are affecting the global burden of dermatological infections and there is a lack of information on the association between climatic factors and MRSA infections. Therefore, association of temperature and relative humidity (RH) with occurrence of SA-SSTIs (*n* = 387) and also MRSA (*n* = 251) was monitored for 18 months in the outpatient clinic at a tertiary care hospital located in Bhubaneswar, Odisha, India. The Kirby-Bauer disk diffusion method was used for antibiotic susceptibility testing. Time-series analysis was used to investigate the potential association of climatic factors (weekly averages of maximum temperature, minimum temperature and RH) with weekly incidence of SA-SSTIs and MRSA infections. The analysis showed that a combination of weekly average maximum temperature above 33 °C coinciding with weekly average RH ranging between 55% and 78%, is most favorable for the occurrence of SA-SSTIs and MRSA and within these parameters, each unit increase in occurrence of MRSA was associated with increase in weekly average maximum temperature of 1.7 °C (*p* = 0.044) and weekly average RH increase of 10% (*p* = 0.097).

## 1. Introduction

It is being realized more and more that the phenomenon of climate change is increasing the global burden of infectious diseases [1,2]. The peak seasons of many dermatological infections, particularly of those that are highly sensitive to temperature and humidity, are getting altered in recent times [3,4,5,6]. Understanding the impact of climatic factors on the incidence of skin and soft-tissue infections is therefore an emerging issue [1,3].

*Staphylococcus aureus* are Gram-positive bacteria that are one of the major causes of skin and soft tissue infections (SSTIs) in all age groups [7,8]. The skin and soft tissue infections caused by *S. aureus* (SA-SSTIs) have demonstrated a significant surge in temperate and tropical settings during the warm months of the year [9]. There is also evidence of a seasonal effect on the incidence of community associated methicillin-resistant *Staphylococcus aureus* (CA-MRSA) infections [3,9,10]. CA-MRSA infections can be difficult to treat because of antibiotic resistance and their rate of infections is increasing rapidly throughout the world [7,8,11,12].

*S. aureus* infections are more common in tropical countries [13,14] like India. The factors that contribute towards the higher rate of SSTIs in tropical countries are overcrowding, poor hygiene, limited water availability and hot and humid weather conditions [14]. In our previous qualitative studies, both community members and healthcare professionals perceived that skin infections are associated with climatic factors [15,16]. Furthermore, we also found that biophysical environment is associated with antibiotic resistance [17]. Since studies on environmental epidemiology of *S. aureus* are relatively few in India [9,14] and as, antibiotic resistance including MRSA is a major public health concern in India [18,19]; we investigated the association of temperature and relative humidity (RH) with the occurrence of SSTIs, SA-SSTIs and MRSA. Most of the earlier studies are retrospective, analyzing historical data for association of *S. aureus* infections. Considering this and also taking into account the fact that like in many other low and middle income countries, verifiable clinical or hospital records are generally not available in India, we decided to conduct a prospective study in which we clinically and microbiologically verified *S. aureus* infections as well as MRSA and analyzed their association with local temperature and RH data.

## 2. Methods

### 2.1. Study Design and Setting

This prospective study was conducted from July 2009 to December 2010 at Kalinga Institute of Medical Sciences (KIMS), Odisha, India. KIMS is a 500-bed tertiary care teaching hospital located in Bhubaneswar, the capital of the state of Odisha. The city has a tropical climate.

### 2.2. Study Participants and Sample Collection

We prospectively enrolled consecutive patients clinically diagnosed to have SSTIs from the outpatient clinic of the Department of Dermatology and Surgery of KIMS, Bhubaneswar. Patients with the following SSTIs were included: impetigo, furuncle, carbuncle, cellulitis, pyoderma and erythrasma. Patients having a SSTI severe enough to require hospitalization were not included in the study. Study assistants collected a pus swab from the SSTI site of consecutive patients. Only one sample per patient was included. The samples were transported in Amies transport media with charcoal on ice and reached the Microbiology Laboratory of the KIMS hospital for further analysis within two hours of collection. Informed consent was obtained from the participants for sample collection after explaining the purpose of the study. The ethical committee of the KIMS approved the study.

### 2.3. Isolation of S. aureus and Antibiotic Susceptibility Testing

The pus swabs were inoculated onto blood agar plates. The plates were incubated at 35 °C for 24 to 48 h. Colonies of *S. aureus* were confirmed by their typical morphology using Gram’s staining, anaerobic utilization of glucose and mannitol, catalase production and tube coagulase test [20].

The Kirby-Bauer disk diffusion method was used for antibiotic susceptibility testing. The antibiotic disk strengths were as per Clinical and Laboratory Standard Institute (CLSI) guidelines at the time of the study [20]. Cefoxitin disk screen test and 6 µg/mL of oxacillin in Mueller-Hinton agar supplemented with 4% NaCl were used for screening of methicillin resistance. *S. aureus* ATCC 25923 was used as control strain. The panel of antibiotics was selected based on local antibiotic prescription patterns and as per the CLSI guidelines at the time of the study [20]. Antibiotic susceptibility testing of *S. aureus* isolates was done for the following antibiotics: oxacillin (1 µg), ampicillin/sulbactam (10/10 µg), ceftriaxone (30 µg), erythromycin (15 µg), amikacin (30 µg), ciprofloxacin (5 µg), vancomycin (30 µg), and linezolid (30 µg).

### 2.4. Climate Data

The records of maximum and minimum temperature in degrees Celsius (°C) and relative humidity in percentage (%) at 08:30 h and 17:30 h were obtained from the meteorological station at Bhubaneswar. We obtained daily records for the duration of the study *i.e.*, from 1 July 2009 to 31 December 2010. Average weekly mean of maximum and minimum temperatures and RH were calculated from the daily records.

### 2.5. Statistical Analysis

Weekly data were analyzed. Two-way line plots, uniformly weighted moving averages (using two lagged terms, two forward terms, and the current observation) and order 4 polynomial trends were used to estimate the general trend of the considered outcomes (SSTIs, SA-SSTIs and MRSA) over the study period. Restricted cubic splines with three knots and 95% confidence intervals (CI) were used to study the potential association of climatic factors (averages of weekly; maximum temperature, minimum temperature, and RH) with the study outcomes.

Three regression models with Newey-West standard errors and coefficients estimated by ordinary least squares (OLS) regression, assuming a heteroskedastic error structure and a maximum lag to be considered in the autocorrelation structure equal to 2, were used to study the relationship between the outcomes and the cubic splines of independent variables. The cubic splines knots were chosen on the basis of the cut-off evident from the graphs. *p*-values less than 0.05 were considered significant in the regression models; *p*-values between 0.05 and 0.10 were considered as of “borderline significance”. Analyses were performed using Stata 12 software (Stata Corp. College Station, TX, USA).

## 3. Results

Pus samples from a total of 590 patients with SSI were collected during the study period. Out of these samples 387 (66%) were found culture positive for *S. aureus* and among them 251 (65%) isolates were MRSA. The median and range for weekly samples were: SSTIs (7, 1–17), SA-SSTIs (4, 0–14) and MRSA (3, 0–11). The temperature and RH recorded during different seasons are given in Table 1. During the study period, the maximum temperature ranged between 26 and 41 °C, minimum temperature between 13 and 29 °C and RH between 55% and 97 %.

**Table 1:** Description of climatic factors * in various seasons at Bhubaneswar, India.

Seasons	Climatic Factors (Weekly Averages (Range))		
	Maximum Temperature in °C	Minimum Temperature in °C	Relative Humidity in %
Early summer (mid-February to mid-April)	34–41	18–28	55–76
Late summer (mid-April to mid-June)	33–40	25–29	64–81
Early monsoon (mid-June to mid-August)	29–36	25–27	80–97
Late monsoon (mid-August to mid-October)	30–33	22–27	73–95
Early winter (mid-October to mid-December)	26–33	14–24	63–87
Late winter (mid-December to mid-February)	28–32	13–18	57–74

* Based on climatic records from 1 July 2009 to 31 December 2010 of Bhubaneswar.

Table 2 shows a fitted regression model for the effect of temperature and relative humidity on the incidence of SSTIs, SA-SSTIs and MRSA.

**Table 2:** Fitted regression model * for the effect of temperature and relative humidity on the incidence of the skin and soft-tissue infections (SSTIs), skin and soft-tissue infections caused by *S. aureus* (SA-SSTIs) and methicillin-resistant *S. aureus* (MRSA).

	Weekly Average Maximum Temperature °C						Weekly Average Minimum Temperature °C						Weekly Average Relative Humidity in %					
Cut-off **	Below 33			Above 33			Below 24			Above 24			Below 78			Above 78		
Outcomes	Coef.	95% CI	*p*	Coef.	95% CI	*p*	Coef.	95% CI	*p*	Coef.	95% CI	*p*	Coef.	95% CI	*p*	Coef.	95% CI	*p*
SSTIs	−0.20	−0.97 to 0.56	0.600	0.60	−0.43 to 1.62	0.253	−0.48	−1.19 to 0.22	0.180	0.90	0.04 to 1.76	0.040	0.24	0.03 to 0.45	0.025	−0.25	−0.50 to 0.016	0.065
SA-SSTIs	−0.56	−1.14 to 0.01	0.056	0.69	−0.02 to 1.41	0.058	0.03	−0.69 to 0.77	0.919	−0.12	−0.95 to 0.71	0.781	0.10	−0.06 to 0.27	0.228	−0.17	−0.37 to 0.02	0.078
MRSA	−0.42	−0.86 to 0.03	0.066	0.57	0.01 to 1.12	0.044	0.03	−0.47 to 0.54	0.895	−0.11	−0.68 to 0.48	0.718	0.10	−0.02 to 0.22	0.097	−0.18	−0.32 to −0.04	0.012

* Regression models with Newey-West standard errors and coefficients estimated by ordinary least squares (OLS) regression, assuming a heteroskedastic error structure and a maximum lag 2. *p*-values less than 0.05 were considered as significant and *p*-values between 0.05 and 0.10 as of “borderline significance”; ** The cut-off values for temperature and relative humidity were chosen from the graphs of the cubic splines. Coef. = coefficient, negative sign in the coefficient indicates decreasing number of cases and positive sign indicates increasing number of cases.

**Figure 1:**
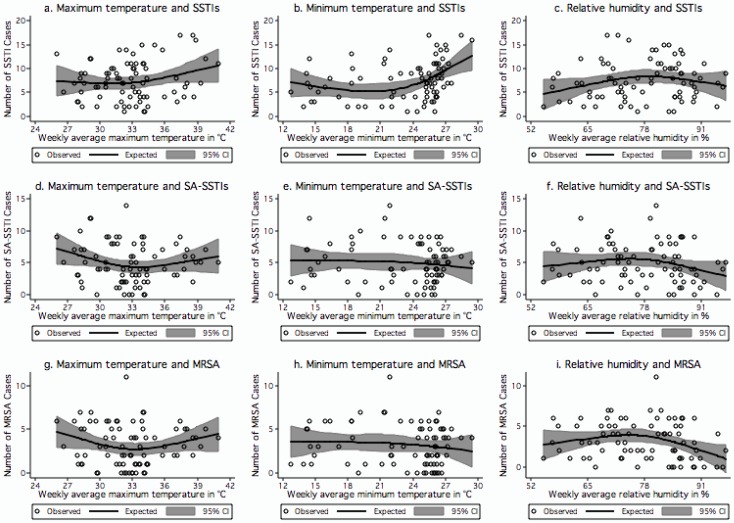
(**a**–**i**) Relationships between climatic factors and skin and soft-tissue infections (SSTIs), skin and soft-tissue infections caused by *S. aureus* (SA-SSTIs) and *SSTIs caused by* MRSA. (**a**) Weekly average maximum temperature and SSTIs; (**b**) Weekly average minimum temperature and SSTIs; (**c**) Weekly average relative humidity and SSTIs; (**d**) Weekly average maximum temperature and SA-SSTIs; (**e**) Weekly average minimum temperature and SA-SSTIs; (**f**) Weekly average relative humidity and SA-SSTIs; (**g**) Weekly average maximum temperature and MRSA; (**h**) Weekly average minimum temperature and MRSA; and (**i**) Weekly average relative humidity and MRSA. The centre lines in the graphs show the estimated spline curve, and the upper and lower lines represent the 95% confidence limits.

Figure 1(a–c) show the variation in the number of SSTI cases in relation to weekly averages of maximum temperature, minimum temperature and RH respectively. The number of SSTI cases increased significantly together with the minimum temperature when its weekly average was higher than 24 °C (coef. 0.90, *p* = 0.04). There was also a significant increase in SSTI cases when the weekly average RH increased from the minimum value of 55% to 78% (coef. 0.24, *p* = 0.025). A borderline significant decrease in SSTI cases was found when the weekly average relative humidity increased above 78% (coef. −0.25, *p* = 0.065). Each unit increase in occurrence of SSTI was associated with minimum temperature increase of 1.1 °C (*p* = 0.04) and RH increase of 4.2% (*p* = 0.025) up to 78%, thereafter each unit decrease was associated with 4% (*p* = 0.065) decrease in RH.

Figure 1(d–f) show the association of number of SA-SSTI cases with weekly averages of maximum temperature, minimum temperature and RH respectively. We found a borderline significant negative relationship of SA-SSTI cases (*i.e.*, the number of cases decreased) with weekly average maximum temperature below 33 °C (coef. −0.56, *p* = 0.056) and a borderline significant positive relationship (*i.e.*, the number of cases increased) with weekly average maximum temperature above 33 °C (coef. 0.69, *p* = 0.058). A borderline significant negative relationship (*i.e.*, the number of SA-SSTIs cases decreased) was observed when the weekly average relative humidity was higher than 78% (coef. −0.17, *p* = 0.078). Within these parameters, each unit increase in occurrence of SA-SSTI cases was associated with a maximum temperature increase of 1.4 °C (*p* = 0.058) and each unit decrease with 5.9% (*p* = 0.078) of RH above 78%.

The association between the number of MRSA cases, and weekly average maximum temperature, minimum temperature and relative humidity is presented in Figure 1(g–i). We observed a borderline significant negative relationship (*i.e.*, the number of cases decreased) between MRSA and weekly average maximum temperature when below 33 °C (coef. −0.42, *p* = 0.066) and the number of cases significantly increased together with the weekly average maximum temperature when above 33 °C (coef. 0.57, *p* = 0.044). There was no significant effect of weekly average relative humidity on number of MRSA cases until RH 78%. The number of MRSA cases significantly decreased when the relative humidity increased above 78% (coef. −0.18, *p* = 0.012). Within these parameters, each unit increase in occurrence of MRSA was associated with maximum temperature increase of 1.7 °C (*p* = 0.044) and RH increase of 10% (*p* = 0.097) up to 78% then there was a unit decrease with 5.5% (*p* = 0.012) decrease in RH.

The antibiotic resistance pattern of *S. aureus* causing SSTIs is shown in Table 3. We found *S. aureus* resistant to erythromycin (79%), ceftriaxone (71%) and ciprofloxacin (61%). The MRSA isolates showed resistance to erythromycin (98%), ceftriaxone (97%) and ciprofloxacin (69%). The methicillin-sensitive *Staphylococcus aureus* (MSSA) isolates showed resistance to erythromycin (44%) and ciprofloxacin (46%). All isolates were susceptible to vancomycin and linezolid.

**Table 3:** Antibiotic resistance pattern of *S. aureus* causing skin and soft-tissue infections.

Antibiotics	*S. aureus*	MSSA	MRSA
	*N* = 387 (100%)	*N* = 136 (35%)	*N* = 251 (65%)
Oxacillin	251 (65)		
Ampicillin/sulbactam	143 (37)	14 (10)	129 (51)
Ceftriaxone	276 (71)	33 (24)	243 (97)
Erythromycin	306 (79)	60 (44)	246 (98)
Amikacin	183 (47)	26 (19)	157 (63)
Ciprofloxacin	236 (61)	62 (46)	174 (69)
Vancomycin	0 (0)	0 (0)	0 (0)
Linezolid	0 (0)	0 (0)	0 (0)

## 4. Discussion

To the best of our knowledge, this is the first prospective study that has investigated the relationship between actual local maximum temperature, minimum temperature and relative humidity with SA-SSTIs and MRSA using time-series analysis. The findings generally show that an average weekly maximum temperature above 33 °C coinciding with an average weekly relative humidity between 55% and 78%, is a favorable combination for the occurrence of *S. aureus* associated skin infections as well as MRSA infections. This combination of temperature and relative humidity is observed during late summer (mid-April to mid-June) in Bhubaneswar, where the year is locally divided into six seasons (Table 1).

A study in New Delhi, India [21] showed bacterial skin infections occurring more frequently in late summer and early monsoon. A study from Pondicherry, India also found higher SSTIs during summer [22]. Similar findings regarding seasonality of *S. aureus* skin infections have been reported from several parts of the world, like the United Kingdom [23], the Netherlands [24], Nigeria [25] and USA [9]. However, these studies link the relationship with months or seasons and not to the actual temperatures existing locally as is done by us.

There is a complex relationship between environment and bacterial growth. Survival and growth of the *S. aureus* bacteria on a host depends on the components of the environment especially temperature, humidity, exposure to sunlight, pH and salinity [26]. The colonization of *S. aureus* increases with hydration of the stratum corneum of the skin [27]. The process of hydration peaks when both environmental temperature and relative humidity are high, which promote sweat production [8,9]. In our data we find that minimum and maximum temperatures both peak in late summer *i.e.*, mid-April to mid-June, however the peak humidity is observed in early monsoon *i.e.*, mid-June to mid-August (Table 1). With the arrival of monsoon the temperatures drop down as the humidity peaks. In the late summer season with highest minimum and maximum temperatures, the RH observed is probably high enough to promote growth of *S. aureus* on human skin. Our finding shows that the combination of average weekly maximum temperature above 33 °C and average weekly relative humidity between 55% and 78% which occurred during mid-April to mid-June increased the occurrence of *S. aureus* associated skin infections and MRSA infections. Wang *et al.* [28] in a time series analysis study of retrospective data of four years (2005–2008) from Arizona, USA, reported a significant correlation between mean monthly temperature and mean monthly specific humidity and incidence of *S. aureus* infections.

Since, humans are primary reservoir of *S. aureus* infection and person-to-person transmission the main route of transmission [29], it is postulated that person-to person transmission increases in the warm season. The local skin temperature and environmental humidity are most conducive for growth of *S. aureus* in late summer and early monsoon in India. Previous studies from India [21,22] have shown that, in addition to hot and humid weather, poor socioeconomic status, overcrowding and poor standards of hygiene are associated with SSTIs.

MRSA infections have earlier been reported to be increasing in India [19,30]. A study conducted in a tertiary care rural hospital in central India found 51.8% MRSA among hospital-associated infections [31]. In our study, we found that as high as 65% of SA-SSTIs were caused by MRSA and it must be noted that our study was on patients with community-acquired infections. Outpatient antibiotic use is an important determinant of antibiotic resistance. In India a myriad of factors can lead to irrational and over prescription of antibiotics [18,32] including the availability and sale of over the counter antibiotics. Although MRSA prevalence was high in our study, a redeeming feature was that we found no resistance to vancomycin or linezolide, the last resort antibiotics, an observation which is similar to results from other studies from India [30,33].

In countries like India, socio-behavioral environmental factors accentuate the effect of climatic factors on bacterial survival and colonization contributing to increased risk of infections at increased temperatures. These factors, amongst others, can be for example, (a) places of overcrowding, which accentuate the effect of high temperature and humidity resulting in profuse sweating creating favorable condition for proliferation of bacteria; (b) limited water availability that results in poor hygiene, a consequence of which is perpetuation of infections and also transmission of them to others and; (c) also poor hygiene habits by themselves amongst local populations [14]. To this can also be added a fact that livestock are an integral part of the household environment in rural India. Livestock associated MRSA occurring in human contacts, is a potential public health problem, the magnitude and implications of which have not been fully understood in India [34]. One additional factor could also be seasonal fluctuations in antibiotic prescribing rates that may have an impact [32].

Our study highlights that physicians in India and in geographical regions having similar climatic characteristics should be mindful of effect of climate factors on the epidemiology of SSTIs caused by *S. aureus* and manage their patients accordingly, both in terms of prevention and treatment of community associated *S. aureus* infections. In late summer and early monsoon physicians should emphasize on personal hygiene measures like regular bath, hand hygiene with soap and water or using of alcohol based hand rub and avoiding reuse or sharing of personal items like razors, towels *etc.* that have contacted infected skin [35]. Patients should be advised to keep fingernails trimmed and avoid picking skin when having SSTI [35]. Physicians should also consider nasal and topical decolonization strategies of asymptomatic household contacts during the high transmission season [35]. Hospitals in similar climatic conditions can consider screening for *S. aureus* for nasal and/or groin carriage during the high transmission season [36].

The main strength of our study is that we prospectively collected the data simultaneously clinically and microbiologically confirming *S. aureus* and MRSA infections, and conducted a time series analysis of the infection data with climatic factors, which has not been done earlier. Previous published literature on the issue of seasonality of *S. aureus* infections is marred by lack of appropriate study design and inadequate emphasis on meteorological factors [10].

This study is the first from India that demonstrates using time-series analysis, the effect of climate variables (temperature and RH) on the epidemiology of SA-SSTIs and MRSA. To our knowledge, this is also a rare study regarding investigation of association of temperature and humidity with MRSA, where confirmation of *S. aureus* infection and methicilin resistance was obtained clinically and microbiologically and the data was analyzed using time-series analysis on weekly basis. Our study has some limitations. We collected data from a single center only, but the advantage of this was that it was a cohesive community sample. Also we did MRSA screening by cefoxitin disk screen test. The molecular method (test for the *mecA* gene) for confirmation could not be performed due to financial constraints.

## 5. Conclusions

Our study shows that a maximum temperature above 33 °C (up-to 41 °C in this study) coinciding with a relative humidity between 55% and 78% is a favorable condition for the occurrence of *S. aureus* associated skin infections and MRSA infections. Since 65% of *S. aureus* infections in our study were MRSA, we propose that the combination of temperature above 33 °C (up-to 41 °C in this study) and a relative humidity between 55% and 78% might also have a conducive impact on MRSA skin infections.
